# Study of patients' attitude to automatic interpretation of laboratory test results and its influence on follow-up rate

**DOI:** 10.1186/s12911-022-01805-w

**Published:** 2022-03-27

**Authors:** Georgy Kopanitsa

**Affiliations:** ITMO University, 4 Birzhevaya Liniya, Saint-Petersburg, Russia

**Keywords:** Laboratory test, Interpretations, Clinical decision support systems, Follow-up

## Abstract

**Background:**

One of the current major factors of not following up on the abnormal test results is the lack of information about the test results and missing interpretations. Clinical decision support systems (CDSS) can become a solution to this problem. However, little is known how patients react to the automatically generated interpretations of the test results, and how this can affect a decision to follow up. In this research, we study how patients perceive the interpretations of the laboratory tests automatically generated by a clinical decision support system depending on how they receive these recommendations and how this affects the follow-up rate.

**Methods:**

A study of 3200 patients was done querying the regional patient registry. The patients were divided into 4 groups who received:

1. Recommendations automatically generated by a CDSS with a clear indication of their automatic nature.
2. Recommendations received personally from a doctor with a clear indication of their automatic nature.
3. Recommendations from a doctor with no indication of their automated generation.
4. No recommendations, only the test results.

A follow-up rate was calculated as the proportion of patients referred to a laboratory service for a follow-up investigation after receiving a recommendation within two weeks after the first test with abnormal test results had been completed and the interpretation was delivered to the patient. The second phase of the study was a research of the patients’ motivation. It was performed with a group of 789 patients.

**Results:**

All the patients who received interpretations on the abnormal test results demonstrated a significantly higher rate of follow-up (71%) in comparison to the patients who received only test results without interpretations (49%). Patients mention a time factor as a significant benefit of the automatically generated interpretations in comparison to the interpretations they can receive from a doctor.

**Conclusion:**

The results of the study show that delivering automatically generated interpretations of test results can support patients in making a decision to follow up. They are trusted by patients and raise their motivations and engagement.

**Supplementary Information:**

The online version contains supplementary material available at 10.1186/s12911-022-01805-w.

## Background

The primary goal of clinical decision support systems (CDSSs) is to provide relevant information to the stakeholders where and when it is needed. These systems provide knowledge, models, and data processing tools to help the experts, doctors and patients make better decisions in clinical situations [1].

Most of CDSSs are intended to be used by doctors. They link health observations with health knowledge to influence health choices by clinicians for improved health care [2]. Nowadays, application of clinical decision support systems (CDSS) by patients is growing fast. They can help patients in different clinical conditions [3]. Patient facing systems provide patient-specific alarms, reminders, clarifications, and recommendations facilitating better healthcare delivery [4, 5].

There are successful implementations of the patient facing CDSS for the interpretation of laboratory test results [6], vital signs alerts [7], dietary helpers [8, 9], and many other clinically relevant support systems.

Provision of a direct access to the results of laboratory test to the patients can make them better-informed [1], more motivated and engaged [10], and able to make informed decisions and better manage their health [11]. Patient safety is another aspect that can be improved by the decision support systems. According to Casalino et al. patients do no follow up the abnormal test results in up to 26% of the cases [12]. Access to the results of laboratory tests is valued by patients and improves motivation and adherence. However, according to Pillemer et al. this may cause nervousness of patients and increase the amount of visits to doctors [13]. Sung et al. supported this by expressing a major concern [14] that direct access to the results may trigger unnecessary nervousness because patients can experience complications when they try to interpret the results. Direct access to the test findings may let patients pursue a proper follow-up on the laboratory tests. Interpretations given by a CDSS can support patients as they proved to deliver correct recommendations [6]. If CDSS could provide timely and high-quality interpretations of laboratory test results to patients, this would support patients to make an informed decision how to continue the diagnostics. This can be especially important in case of abnormal results and in the setting when a patient can’t visit a doctor. This case is particularly important in developing countries.

However, there still is the question of how the patients trust automatically generated decisions in comparison with those given by their doctors and how this affects their decision to follow up on the tests.

In this study we research the influence of automatically generated interpretations of abnormal test results and compare this with the follow-up rate after a doctor’s visit.

The goal of this study is to analyze if the automatically generated interpretations of the laboratory test results can influence a decision to follow up and how patients perceive the automatically generated interpretations.

## Methods

### Description of the CDSS

A clinical decision support system [15], which was used in the study is a patient oriented system that provides interpretations of laboratory test results (Table 1). The CDSS is not certified as a medical device. There is no certification scheme yet in Russia. The clinical validation was done and published previously [15].

### Patient selection

The regional patient registry of the Tomsk region stores all laboratory test orders and results for every patient. This registry is a regional part of a national electronic health infrastructure. It operates since 2014. The purpose of the reginal registry is to provide access to the results of laboratory test results to the healthcare providers in the region and export medical data to other regions on request. By the time of the study begin, the registry contained data of 837,487 patients. We queried only general practitioner patients 18 -76 years old who had their laboratory tests with available automatic interpretation done between September and December 2018.

*The inclusion criteria were:*

- Age: 18–76 years old
- Medical profile of the first encounter: General practitioner
- Available automatic interpretation
- Time interval: 1st of September 2018–31st of December 2018
- Diagnosis: one of the diagnosis or condition mentioned in the Table 7
- Laboratory test: one of the six most done laboratory tests (Table 6)
- At least one abnormal test result

*The exclusion criteria were:*

- Age: < 18 or > 76
- Time interval: outside of 1st of September 2018–31st of December 2018
- Not available automatic interpretation
- Diagnosis not in the list of most common (Table 7)
- Laboratory test outside of the most common list (Table 6)
- No abnormal test results

53,628 patients met these inclusion or exclusion criteria.

A study of 3200 randomly selected patients aged 18–76 in 14 state clinics and two private laboratory services was done querying the regional patient registry. One clinical case of one patient could be included in the study.

**Table 1:** Example of complete blood count results

Complete blood count		
Parameter	Value	Reference interval
Leukocytes (WBC)	4.42 *10^9/l	4.00–10.00
Erythrocytes (RBC)	3.93 *10^12/l	3.80–5.80
Hemoglobin (HGB)	151 g/l	126–174
Hematocrit (HCT)	42.6%	37.0–51.0
Mean corpuscular volume (MCV)	83.0 fL	81.0–102.0
Mean corpuscular hemoglobin (MCH)	28.7 pg	27.0–34.0
Mean corpuscular hemoglobin concentration (MCHC)	310 g/l	300–380
Platelets (PLT)	66 *10^9/l	180–320

### Group assignment

The patients were eligible for the study only with a limited list of diagnoses or conditions that were the most common (76.8% in 2017) in the region and required laboratory tests. The clinical conditions are presented below with their 10th revision of the International Statistical Classification of Diseases and Related Health Problems (ICD-10) with the relations to the laboratory tests:

1. General medical exam (Z00.01)
  - Complete Blood Count
  - Urinalysis
  - Prothrombin Time
2. Essential (primary) hypertension (I10)
  - Complete Blood Count
  - Urinalysis
  - Lipid Panel
3. Cardiovascular disease (I25.X)
  - Lipid Panel
  - Complete Blood Count
4. Diabetes (E10 Type 1 diabetes mellitus and E11 Type 2 diabetes mellitus)
  - Complete Blood Count
  - Urinalysis (UA)
  - Basic Metabolic Panel
5. Hypothyroidism (E03.9)
  - Thyroid Stimulating Hormone
  - Basic Metabolic Panel
6. Iron deficiency anemia (D50.9)
  - Complete Blood Count
  - Iron panel
7. Glomerulonephritis (N00.9)
  - Complete Blood Count
  - Urinalysis
8. Hypoglycemia (E16.2)
  - Complete Blood Count
  - Urinalysis
  - Basic Metabolic Panel

Please see the Additional file 1 for the details of the panels.

For each diagnosis or condition, we measured the ratio of tests and the follow-up rate.

Six most done laboratory tests (86.4% of all test orders in 2018) were selected for the study. For all these tests the CDSS was able to produce interpretations that included a recommendation to have another laboratory test or to do the same test again. A recommendation depends on the clinical status of the patient. The most common follow-up recommendations for each of the six laboratory tests are presented in the Table 2.

**Table 2:** Common follow-up recommendations for the laboratory tests

Test	Recommendations to run a test	Possible diagnosis
Complete blood count	Prothrombin time Basic metabolic panel Liver panel Lipid panel Iron panel Peripheral blood smear Lactate dehydrogenase	Hemolytic anemia (D59) Iron deficiency anemia (D50)
Urinalysis	Glycated Hemoglobin A1C Random blood sugar test Albumin to creatinine ratio C-peptide Complete blood count Basic metabolic panel	Diabetes mellitus (E8–E12) Kidney disease
Prothrombin time	Platelet count Platelet function tests, Coagulation factor tests	Vitamin K deficiency (E56.1)
Basic metabolic panel	Glycated Hemoglobin A1C test Random blood sugar test C-peptide test Comprehensive metabolic panel	Diabetes mellitus (E8-E12) Kidney disease
Lipid panel	lipid panel	Cardiovascular disease I25.X
Thyroid stimulating hormone	T4 thyroid hormone tests T3 thyroid hormone tests	Hypothyroidism E03.9 Hyperthyroidism E05.90

Only patients with at least one abnormal test result were included in the study. The reference intervals for the patients were taken from the recommendations of the Ministry of Health of the Russian Federation. We considered test results to be abnormal only if they were at least 20% outside of the reference interval because it indicated a danger or potential risk for the patient's well-being over time. In case of more than 1 abnormal test the CDSS considered the combination of abnormal values and generated the interpretations accordingly.

The study included 4 groups of patients:

1. The interpretations for the group 1 were sent automatically per email. They contained a clear indication that they were generated automatically.
2. The interpretations for the group 2 were given by a doctor during a visit. They contained a clear indication that they were generated automatically. A special flag was introduced to track the fact that the automatically generated interpretation was delivered to the patient by a doctor. The doctors were instructed how to use this feature prior to the study begin.
3. The interpretations for the group 3 were given by a doctor during a visit. They contained no indication that they were generated automatically. A special flag was introduced to track the fact that the automatically generated interpretation was delivered to the patient by a doctor. The doctors were instructed how to use this feature prior to the study begin.
4. The patients from the group 4 received only the results of laboratory tests per email with reference intervals without any interpretations

A summary of the groups is given in the Table 3.

**Table 3:** Summary of the patients’ groups

Group	Automatic interpretation mark	Source
Group 1	Clear indication	Automatic notification
Group 2	Clear indication	Doctor
Group 3	No indication	Doctor
Group 4	No interpretation	No

For the groups 2 and 3 the CDSS system randomly selected to put and automatic interpretation indication on the test results.

To assign the patients to the group 1 a patient had to meet 1 condition:

- No doctor’s visit was registered within 2 weeks after the test result were sent to the patient.
  To assign the patients to the group 4 a patient had to meet the following condition:
- No doctor’s visit was registered within 2 weeks after the test result were sent to the patient.

Patients were assigned to each group by a Random Sampling method. We ensured the comparability to Russian demographic distributions during the random sampling by providing constraints to the sampling script. The assignment was made automatically according to the following algorithm:

1. The assignment system receives a notification on the completed laboratory test with abnormal results
2. The system checks the inclusion and exclusion criteria
3. If the patient meets the inclusion or exclusion criteria, the system does a random assignment to one of the groups. To ensure the comparability to Russian demographic distributions during the random sampling the script considered demographic constraints. In case the patient doesn’t meet the demographic constraints of the group it is unassigned and returned to the patient pool.
4. The system sends the interpretation to the group 1 patients and test results without interpretations to the group 4 immediately after the assignment.
5. The system increments a counter for a corresponding group.
6. For the group 2 and 3 the system generates interpretations that can be queried by a hospital information system (HIS) during the appointment.
7. The system increments the counters for the groups 2 and 3, only after a notification that a patient has received an interpretation from a doctor
8. The process runs until counters for each of the groups reach 800.

All the doctors from the participating clinics were instructed how to work with the automatically generated interpretations before the study begin. As the clinics already had at least 2 years of experience of working with automatic interpretations the doctors were instructed how to work with different types of interpretation reports for the different groups of patients. In particular, the doctors we taught how to identify a patient group and to act with the interpretations for the groups 2 and 3. For the group 2 the doctors were asked to point that the interpretations were done automatically during the discussion of the results with the patients. For the group 3 the doctors were asked not to mention the automatic nature of the interpretations during the discussion of the results with the patients.

**Fig. 1:**
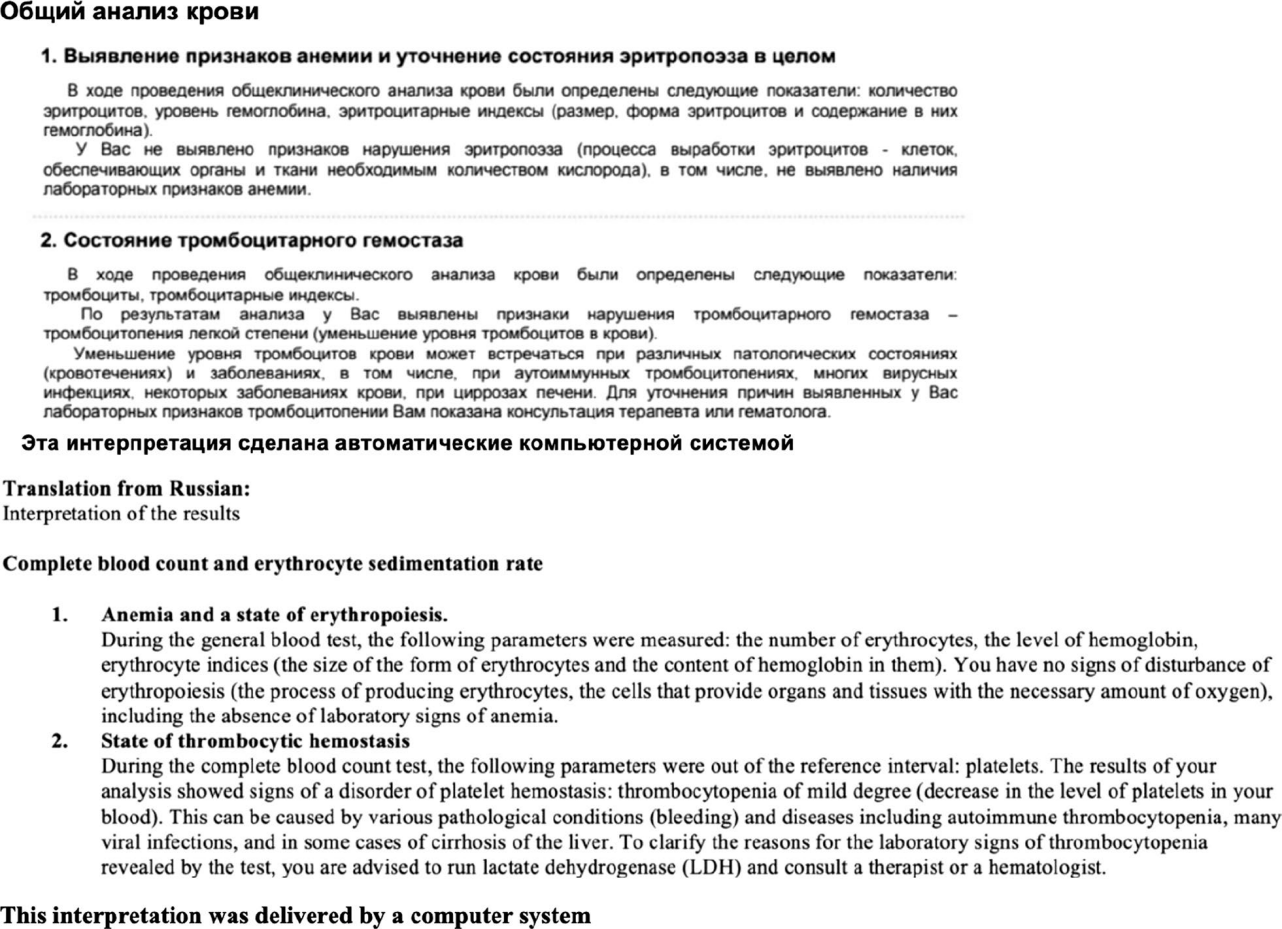
Example of an interpretation

The interpretations provided to the patients included a table with the results (see Table 1 for an example) and a text in Russian with a recommendation (see Fig. 1 for an example in Russian and an English translation).

### Statistical analysis

We calculated a follow-up rate with 95% two-sided confidence interval as the proportion of patients who performed a follow-up visit to a doctor or ordered a laboratory test after they have received an interpretation with a recommendation.

We considered only the patients who referred to a laboratory service or a doctor during two weeks after they have received abnormal test results with interpretations.

We calculated follow-up rates separately for the ≥ 60 years old and < 60 years old patients as 60 is the age when people are considered senior citizens in Russia.

The chi-square statistic tests were done between groups 1 and 4 and groups 2 and 3 to test the statistical significance of the results.

### Attitude survey

The patients from the groups 1 and 2 were asked to participate in a survey to study their motivation to follow up or not once they have received an interpretation with a recommendation. We focused on the role of the interpretations that were generated automatically. We used emails and personal telephone calls to invite the patients. Only the patients 18 years and older were eligible for the study.

All the patients from the group 1 and group 2 of the initial study were invited for the interviews. 836 patients accepted the invites: 421 of them from the group 1 and 415 from the group 2. We established a study cohort of 789 patients that represented the initial study demographic distribution considering the age and sex restraints.

The study was set up as a series of personal structured interviews. The questionnaire was developed and validated [16] by the study team and approved by the local ethics committee (Additional file 2). The patients were requested to rate questions 2–5 using a 7 points Likert scale [17]:

1. (Strongly disagree),
2. (Disagree),
3. (Somewhat disagree),
4. (Neither agree or disagree),
5. (Somewhat agree),
6. (Agree),
7. (Strongly Agree). The question 1, was a yes/no question. The study team did the interviews by phone or in person. Each of them by one interviewer. Each interview took about 5 min. All interviews were recorded, and the data was later transcribed by the research team.

After the interview agenda was prepared by the project team it was reviewed and approved by the local ethics committee of the Tomsk Region. Before the start of every interview, we informed the participants about the interview and research purpose. Every interviewee has received declaration of anonymity and confidentiality in printing. Each participant signed and submitted a written consent before the start if the interviews. The interviews were conducted in February–March 2019. We used a Russian version of the interview agenda. The paper provides an English translation.

The interview plan comprised of the following questions:

1. Did you notice that the recommendation was generated automatically?
  Pease rate from 1 to 7 the following statements:
2. The automatic nature of interpretation of your test results influenced your decision to follow up.
3. Doctors can give more accurate and valid test results’ interpretations
4. I will wait for an interpretation and a recommendation from a doctor instead of getting an automatic interpretation immediately
5. I trust the interpretations that were generated automatically

In addition to these questions, we asked patients about their education using multiple choice questions: Higher, Secondary, or Below secondary. We also evaluated the patients' computer literacy based on how often they use PC or other personal devices. We graded their computer literacy as beginners: users that had started using PC only within our project; intermediate: users who were using a PC at least twice a week; and advanced: for those who were using a PC on the daily basis.

### Ethics approval and consent to participate

The study was approved by the ethics committee of the regional Department of Health of the Tomsk Region. Ethical issues of the secondary use of this registry to identify individual patients and their clinical issues were addressed during the approval of the study. We queried only the anonymized data with IDs, that we could not associate with real people during the first phase of the study. The approval included the access the contact data of the patients for a follow up attitude study. An administrative permission from the regional Department of Health of the Tomsk Region was acquired to access the clinical/personal patient data used in your research.

## Results

**Fig. 2:**
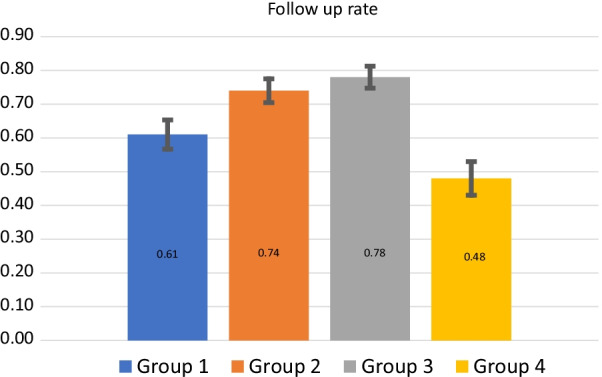
Follow up rate per group

Table 4 presents the demographic characteristics of the study participants that were derived from the regional patient registry. The follow-up rates for the groups 1–4 are presented in Table 5 and Fig. 2.

**Table 4:** Demographic characteristics of the patients

Sex	Group 1		Group 2		Group 3		Group 4		Average age	Total
	≥ 60 19.4%	< 60 80.6%	≥ 60 19.4%	< 60 80.6%	≥ 60 19.4%	< 60 80.6%	≥ 60 19.4%	< 60 80.6%		
Males	47.0%		46.75%		47.38%		46.38%		38.8	46.87%
	74	302	70	304	76	303	72	299		1500
Females	53.0%		53.25%		53.62%		53.62%		39.4	55.13%
	97	327	95	331	97	324	99	330		1700
*Total*	171	629	165	635	173	627	171	629

**Table 5:** Laboratory test follow-up rate for every group

Sex	Group 1,%		Group 2,%		Group 3,%		Group 4,%	
Age	≥ 60	< 60	≥ 60	< 60	≥ 60	< 60	≥ 60	< 60
Males	59 ± 14.6 CI	61 ± 7.1 CI	73 ± 12.2 CI	74 ± 5.7 CI	75 ± 11.7 CI	79 ± 5.7 CI	48 ± 16.7 CI	42 ± 8.6 CI
Females	61 ± 12.4 CI	62 ± 6.8 CI	72 ± 10.6 CI	75 ± 5.4 CI	73 ± 10.3 CI	79 ± 4.9 CI	55 ± 13.2 CI	49 ± 7.7 CI
Total, age-dependent	55 ± 10.1 CI	61 ± 4.9 CI	72 ± 8.3 CI	75 ± 3.9 CI	74 ± 10.2 CI	79 ± 4.9 CI	50 ± 10.6 CI	46 ± 5.7 CI
Total	61% ± 4.3 CI		74% ± 3.5 CI		78% ± 3.3 CI		49% ± 5 CI	
Average follow-up after doctor’s visit			76% ± 2.4 CI

The chi-square statistic test was calculated between groups 1 and 4; and between groups 2 and 3.

The *p* value is < 0.01 for the groups 1 and 4. The result is significant at *p* < 0.05. The *p* value is < 0.08 for the groups 2 and 3. The result is significant at *p* < 0.05. So, the difference in the follow-up rates between groups 2 and 3 is not statistically significant.

A breakdown of the follow-up rates per group and test are shown in the Table 6.

**Table 6:** Frequency of laboratory tests and follow-up rates per test

Laboratory test	Fraction, %	Follow-up rate for groups 1–3, %	Follow up rate for group 4, %
Complete blood count	38.0	63.69 ± 3.12 CI	44.97 ± 5.6 CI
Urinalysis	24.5	73.99 ± 3.55 CI	53.33 ± 6.98 CI
Lipid panel	9.09	74.20 ± 5.8 CI	56.72 ± 11.4 CI
Basic metabolic panel	10.56	63.2 ± 5.9 CI	38.09 ± 10.36 CI
Prothrombin time	10.43	64.01 ± 5.95 CI	55.71 ± 10.7 CI
Thyroid stimulating hormone	7.42	76.27 ± 6.25 CI	57.63 ± 12.6 CI
Total	100	68.04 ± 1.87 CI	49.13 ± 3.46 CI

The follow-up rate for different tests varies between 63.2% (158 out of 250) and 76.27% (135 out of 177) for the groups 1–3 and between 38.09% (32 out of 84) and 57.63% (34 out of 59) for the group 4. We can observe similar trends for both groups with the highest rate for the Thyroid Stimulating Hormone test and the lowest for the Basic Metabolic Panel.

Table 7 presents the distribution of diagnoses and conditions, which were the reasons of a visit to a doctor.

**Table 7:** Diagnoses distribution

Diagnosis	Rate, %
General medical exam	30.4
Essential (primary) hypertension	19.2
Cardiovascular disease I25.X	17.1
Diabetes E10 Type 1 diabetes mellitus E11 Type 2 diabetes mellitus	16.8
Hypothyroidism	8.2
Iron deficiency anemia	4.5
Glomerulonephritis	2.7
Hypoglycemia (E16.2)	1.1

**Fig. 3:**
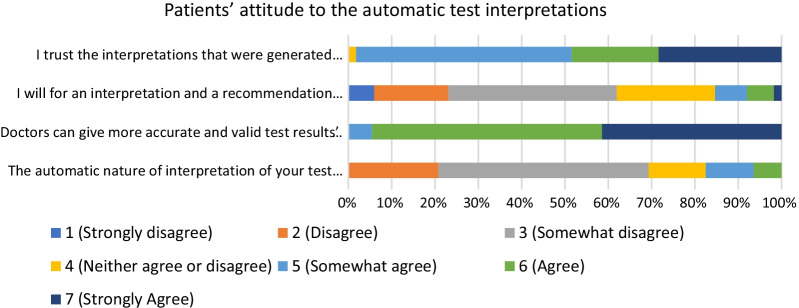
Patients’ attitude to the automatic test interpretations

The study of the attitude and reasons to follow up was performed with 789 patients. The demographic characteristics of the study participants are shown in Table 8. Table 9 and Fig. 3 present the results of the structured interviews.

**Table 8:** The demographic characteristics of the attitude survey participants

Sex	Average age	Age ≥ 60	Education			IT literacy		
			Higher	Secondary	Below secondary	Beginners	Intermediate	Advanced
370 46.9% Males	38.9	70 18.9%	92 24.9%	156 42.2%	122 32.9%	32 8.6%	214 57.8%	124 33.5%
419 53.1% Females	39.2	80 19.1%	95 22.7%	189 45.1%	135 32.2%	71 16.9%	199 47.5%	149 35.6%
Total 789	39.1	160 20.3%	187 23.7%	345 43.7%	257 32.6%	103 13.1%	413 52.3%	273 34.6%

**Table 9:** Patients’ attitude to the automatic test interpretations

Question		789 patients			
		Mean	Median	Max	Min
2	The automatic nature of interpretation of your test results influenced your decision to follow up	3.3	3	6	2
3	Doctors can give more accurate and valid test results’ interpretations	6.4	7	7	5
4	I will wait for an interpretation and a recommendation from a doctor instead of getting an automatic interpretation immediately	3.3	3	7	1
5	I trust the interpretations that were generated automatically	5.8	5	7	4

The mean value for question 1, if the patients noticed at all that the interpretations were done by a computerized system was 75.8% (279 out of 368) for group 1 and 57,9% (244 out of 421) for group 2. This can indicate that the interpretations in the natural language were of a high quality and the patients could not distinguish whether the text was written by a human or a computer. Patients express mildly negative opinion (question 2: mean 3.1–3.4) about the influence of the automatic nature of interpretation on their decisions.

Patients consider time factor as an important advantage of the computer interpretations and are willing to get automatic interpretations if they can receive it faster than the ones from their doctor (question 4: median = 3 out of 7). Discussing the reasons behind the decision to follow up, the patients do trust the computerized clinical decision support systems (question 5: median = 5 out of 7), however, they prefer to receive interpretations and recommendations from doctors (question 3: median = 7). This can be observed from the follow-up rate difference of group 1 (61% (488 out of 800)) and groups 2 (74% (592 out of 800)) and 3 (78% (624 out of 800)).

## Discussion

Patients are mostly prepared and keen to get a direct access to the results of laboratory tests [6]. Automatically produced interpretations and recommendations, particularly when a patient cannot visit a doctor, can improve experience and upsurge motivation to follow-up.

This study reveals how the automatically generated laboratory tests interpretations influence the decision to follow up in case of abnormal results. The findings of the study provide a deeper understanding of the motives to follow up or not after receiving a recommendation and the role of the automatically generated interpretation in this decision.

If we compare the results of group 4 (49% (392 out of 800) follow-up rate) where the patients received only test results without any interpretation with all the patients who received interpretations (groups 1–3 combined) we can see a much higher follow-up rate (average 71% (1712 out of 2400) follow up rate). This emphasizes the value of test interpretations, especially in the healthcare systems where the patients refer directly to the laboratory services without a visit to a doctor and receive only the interpretations produced by laboratory services. For example, in Russia, only about 28% of the patients visit a doctor for laboratory tests [15]. Group 1 (automatic interpretations with no doctor visit) patients have higher follow-up rate (61% versus 49%) than Group 4 (test results with no interpretation). It is important to mention that the follow-up rate for Prothrombin Time was not significantly different (Table 6) between the groups that received recommendations (groups 1–3) and the group that did not receive recommendations (group 4).

The difference in follow-up rates is especially visible in the younger patients: 61% (184 out of 302 for the group 1), 75% (228 out of 304 for the group 2), and 78% (236 out of 303 for the group 3) versus 46% (138 out of 299 for the group 4).

The group 2 (use of CDSS was indicated) has lower follow-up rate (74% versus 78%) than group 3 (no such indication). Although the difference is not statistically significant, the qualitative study (Question 3, Table 9) shows that patients agreed that doctors give more precise and valid interpretation (Mean 6.4 out of 7).

We observed that older people, both males and females, tend to follow up on the lab tests more often than younger people. We observed a significant 11% difference in the follow-up rate of the older patients and younger patients of group 1. This means that older people are more conservative in their attitude towards computerized clinical decision support systems. However, the follow-up rate is of the elder patients of group 1: 55% (94 out of 171) is still higher than of the elder patients of group 4: 49.7% (85 out of 171). In general, sex did not influence the attitude of the patients towards the computerized clinical decision support systems.

Patients still believe that doctors will give better tests interpretations (question 2: median = 7 out of 7).

### Implications of the study

The systematic review by Callen et al. demonstrated that up to 6.8–62% of abnormal laboratory tests are not followed up [18, 19]. Our results have shown that patient facing CDSS for laboratory test interpretations can contribute to solve the issue of failures to notify patients of abnormal outpatient laboratory test results. This is a usual situation currently according to Casalino et al. [12] Up to 26.2% of abnormal laboratory test results are not received and understood by patients. Automatic delivery of laboratory test results along with their interpretations can reduce the amount of uninformed patients.

Our results show the presence of significant impact of automatic interpretations on the decision to follow up on the abnormal laboratory test results. Implementation of notifications on laboratory test result with detailed automatically generated interpretations and recommendations requires integration efforts and improvement of the decision support methods and algorithms to increase their validity and interpretability. This will increase trust to the patient-facing CDSS.

Our results recommend that direct release systems for the patients can be improved by providing clinical interpretation, even made by computerized clinical decision support systems. This can support addressing nervousness related to the receiving of uninterpreted laboratory test results that patients cannot understand, which is one of the major issues of the direct access to test results [20, 21].

Laboratory services in Russia have a legal obligation to provide test results to the patients regardless of whether the patient referred directly to the laboratory or the test had been ordered by a doctor. Providing not only the results but also the interpretations will make these obligatory notifications valuable for patients. This was demonstrated by the results of the research.

There is another point that need to be considered and studied further. Autogenerated interpretations, may deliver much more information to the patients and state many more possibilities than a physician's interpretation. For example, the provided interpretation (Table 1) on low slightly low platelets can have a long list of possible problems including cirrhosis, autoimmune thrombocytopenia, viral infections, etc.

This can potentially have the effect of frightening the patient to seek follow-up, even if not necessarily indicated, especially if they have never seen a comment like this on their lab report before. Thus, a less specific interpretation could show higher follow-up rates than a more targeted and nuanced interpretation. This effect should be further studied.

We didn’t observe any significant correlations between IT literacy and education with the other responses in the attitude survey.

We understand that some of the patients could do a follow-up in a different region and were not included in the study. We did not consider the patients that could follow up in other regions of Russia in this study. The average number of patients receiving medical care outside of the home regions in Russia is 2.7%,^1^ so, we presume that the amount of such patients did not influence the results.

### Limitations of the study

We understand that a CDSS cannot replace a visit to a doctor. It is hard to consider a clinical context and a history of a patient. For example, we would expect that in contrast to a CDSS, physicians are aware of reasons for consultation will characteristically consider the clinical context, when judging on the relevance of readings that are near the border of reference ranges. This is especially significant when a timeline is available. Group 1 and Group 4 were the major groups that can be clearly compared. The doctor's visit groups (2 and 3) could have significant bias due to the patients having more sophisticated discussion with a doctor not limited to the laboratory test results.

The study did not consider the severity of illness as a factor to follow-up. This can have a significant influence on the follow-up rate and requires a further investigation. Our models can consider the timelines; however, they are not available without a registration and maintaining the medical records. For the doctor recent trends might indicate deterioration or recovery, that cannot be considered by a CDSS in most of the cases. When different laboratory tests are conducted simultaneously, various multivariate patterns give rise to plausible interpretations of cause-and-effect. These are the limitations of the CDSS that should be considered and explained to the patients and doctors.

## Conclusions

The results of the study show that delivering automatically generated interpretations of laboratory test results can support patients in making a decision to follow up. They are trusted by patients and raise their motivations and engagement. On the other hand, the results demonstrated that patients still trust interpretations and recommendations that they receive personally from doctors more than automatically generated ones.

## Supplementary Information

**Additional file 1**. Test panels description.

**Additional file 2**. Questionnaire in English.

## Data Availability

The datasets used and/or analyzed during the current study are available from the corresponding author on reasonable request.

## Abbreviations

CDSS: Clinical decision support system
HIS: Hospital information system

## Footnotes

1 https://www.ttfoms.tomsk.ru/page/chislennost_inooblastnyh.
