# Supplementary material for: Study of patients' attitude to automatic interpretation of laboratory test results and its influence on follow-up rate
Source: BMC Med Inform Decis Mak. 2022 Mar 27;22:79. doi: 10.1186/s12911-022-01805-w (PMC8962526; doi:10.1186/s12911-022-01805-w)
Supplement: Supplementary file 1 — Additional file 1. Test panels description. [file 12911_2022_1805_MOESM1_ESM.docx]

Test panels description

**liver panel**

Alanine aminotransferase (ALT)

Serum albumin

Aspartate aminotransferase (AST)

Gamma glutamyl transpeptidase (gamma GT)

Serum iron

Total bilirubin

Direct bilirubin

Total alkaline phosphatase

Total cholesterol

anti-HCV antibodies

HBsAg

alpha fetoprotein (alpha FP)

indirect bilirubin

**lipid panel**

Total cholesterol

Low-density lipoprotein (LDL) cholesterol

Very low-density lipoprotein (VLDL) cholesterol

High-density lipoprotein (HDL) cholesterol

Triglycerides

**basic metabolic panel**

Alanine aminotransferase (ALT)

Aspartate aminotransferase (AST)

Plasma glucose (AST)

Serum creatinine (with determination of GFR)

Serum urea

Serum total protein

Total bilirubin

Total cholesterol

**comprehensive metabolic panel**

Alanine aminotransferase (ALT)

Pancreatic amylase

Aspartate aminotransferase (AST)

Serum total protein

Total bilirubin

Direct bilirubin

Gamma-glutamyl transpeptidase (gamma GT)

Plasma glucose

Serum iron

Serum calcium

Serum creatinine

Leukocyte count

Serum uric acid

Red blood cell sedimentation rate (RBCR) capillary photometry method

Triglycerides

Total alkaline phosphatase

Total Cholesterol

**Iron panel**

Iron, Serum

Iron Saturation

Total Iron Binding Capacity (TIBC)

Unsaturated Iron Binding Capacity (UIBC)

Ferritin
