# Supplementary material for: Study of patients' attitude to automatic interpretation of laboratory test results and its influence on follow-up rate
Source: BMC Med Inform Decis Mak. 2022 Mar 27;22:79. doi: 10.1186/s12911-022-01805-w (PMC8962526; doi:10.1186/s12911-022-01805-w)
Supplement: Supplementary file 2 — Additional file 2. Questionnaire in English. [file 12911_2022_1805_MOESM2_ESM.docx]

Questionnaire

Please answer yes/no to the question 1.

Question 1. Did you notice that the recommendation was generated automatically?

Pease rate from 1 (strongly disagree) to 7 (Strongly agree) the following questions 2-5:

Question 2. The automatic nature of interpretation of your test results influenced your decision to follow up.

Question 3. Doctors can give more accurate and valid test results’ interpretations

Question 4. I will wait for an interpretation and a recommendation from a doctor instead of getting an automatic interpretation immediately

Question 5. I trust the interpretations that were generated automatically
